# Neonatal respiratory support strategies for the management of extremely low gestational age infants: an Italian survey

**DOI:** 10.1186/s13052-019-0639-5

**Published:** 2019-04-11

**Authors:** Flavia Petrillo, Camilla Gizzi, Gianfranco Maffei, Piero G. Matassa, Maria Luisa Ventura, Cinzia Ricci, Roberta Pastorino, Giovanni Vento, on Behalf of Neonatal Pneumology Study Group Italian Society of Neonatology

**Affiliations:** 1 Division of Neonatology, Ospedale Di Venere, Bari, Italy; 2 Azienda Ospedaliera Regionale “San Carlo”, Potenza, Italy; 3 Division of Neonatology, Azienda Ospedaliero-Universitaria “Ospedali Riuniti di Foggia”, Foggia, Italy; 4 Department of Clinical Sciences and Community Health, Fondazione IRCCS Cà Granda Ospedale Maggiore Policlinico, Università degli Studi di Milano, Milan, Italy; 5 Fondazione MBBM - Ospedale San Gerardo Monza, Monza, Italy; 6 Department of Woman and Child Health and Public Health, Division of Neonatology, Fondazione Policlinico Universitario A. Gemelli IRCCS - Università Cattolica del Sacro Cuore, Rome, Italy; 7 Section of Hygiene, Institute of Public Health - Università Cattolica del Sacro Cuore, Rome, Italy

**Keywords:** Survey, Preterm infants, Mechanical ventilation, Non-invasive respiratory support, Training

## Abstract

**Background:**

We aimed to survey Delivery Room and Neonatal Intensive Care Unit (NICU) respiratory strategies dedicated to the extremely low gestational age newborn (ELGAN – GA < 28 wks) in Italy.

**Methods:**

A questionnaire was sent to 113 Italian level III centres. A lead physician and a nurse with expertise in mechanical ventilation (MV) were identified in each unit to answer. Information about those aspects of ventilatory support considered by center’s staff as needing improvement was also collected.

**Results:**

A 100% response rate was obtained. In the Delivery Room, sustained lung inflation was performed in 74.8% of centres, and 89.2% used NCPAP. For ELGANs who need invasive MV, conventional MV was the most used strategy. Volume-targeted ventilation and High-frequency oscillatory ventilation (HFOV) were considered as primary mode in < 30% of centres. Among non-invasive strategies, NCPAP was the most utilized, followed by BiPAP, High-flow nasal cannula and nasal intermittent positive pressure ventilation. Nurses more commonly recorded in the nursing charts the ventilator’s setting parameters rather than measured ones. HFOV and non-invasive ventilation were the most quoted aspects of neonatal ventilation felt as to be improved.

**Conclusion:**

The routine respiratory support practices in Italy showed marked variations among units. Focused interventions are largely required to improve clinical practice.

## Background

Extremely low gestational age newborns (ELGANs), born before 28 weeks of gestation, represent a particularly fragile category of patients, requiring a challenging, careful and multidisciplinary management. Despite the significant progress of the available technologies and modern devices observed in the last few years, ELGANs remain at high risk of developing pulmonary sequelae, especially bronchopulmonary dysplasia (BPD). BPD is a multifactorial disease related to different factors, being ventilator induced lung injury (VILI) the main one. Recent evidences indicate that some ventilatory approaches, such as the “open lung strategy” and the volume-targeted ventilation, are able to reduce VILI and to improve extremely preterm infants respiratory outcomes [1–4]. Nevertheless, there is still a huge heterogeneity among different neonatal intensive care units (NICUs) regarding adopted ventilation strategies and devices. The Working Group on Neonatal Pneumology of the Italian Society of Neonatology strongly promotes the implementation of Best Practice Guidelines on ventilation around the country organizing focused interventions, such theoretical/practical courses and residential trainings, with the aim to meet physicians’ and nurses’ upgrading needs and to improve outcomes in this particular category of newborn babies. Indeed, it has been shown that guidelines implementation is associated with a significant improvement of infants’ respiratory management [5–7].

In 2014, our Working Group performed a national survey among Italian tertiary centres in order to 1) verify the current practice of neonatal respiratory support in DR and NICU in Italy for ELGANs, and 2) gather information about Italian tertiary centres’ staff upgrading needs, and 3) plan, accordingly, tailored residential training or courses on those ventilation strategies considered to be improved in each unit.

## Methods

The survey was conducted between May and September 2014, data referring to 2013. Centers’ participation was voluntary. A lead physician with expertise in mechanical ventilation was identified in each unit by our Working Group who in turn identified an expert nurse. Two different questionnaires, addressed to the lead physician and to the nurse, were emailed. A reminder was sent to non-responders every 10–15 days for a maximum of three times. At that point, if no answer was received, the participant was contacted by phone by an investigator and the questionnaire carried out as a telephonic interview.

Two sessions composed the questionnaire First session asked about Delivery Room (DR) and NICU respiratory support strategies and specific policies adopted for ELGANs. The second session investigated whether there was any area of respiratory support considered as needing improvement in the unit through a local training. Different questions were addressed to physicians and nurses. Physicians were requested to answer on, type of respiratory support provided in the DR, preferred mode of ventilation for invasive and non-invasive respiratory support in NICU, use of volume targeted ventilation, use of techniques of surfactant administration for infants under NIV, such as Intubate-Surfactant-Extubate (INSURE) and Less Invasive Surfactant Administration (LISA) approaches, target FiO_2_ used to deliver surfactant, and administration of analgesic drugs during both MV and surfactant administration in spontaneously breathing infants. Nurses were requested to answer about lower and upper limit of SpO_2_ considered safe in their NICU, type and frequency of respiratory parameters (set and measured) and vital signs recorded on the nursing sheet during invasive and non invasive ventilation, use of Silverman and pain assessment scores, titration of analgesic drug during ventilation and number of patient assigned to each nurse in NICU. Both physicians and nurses were asked to address which strategies needed improvement in their units, with the aim of identifying their specific requests.

## Results

In 2013, 514.308 infants were born in Italy. Of them, 1665 were ELGANs (0.3%). In our country, there are 113 NICUs dedicated to these extremely preterm babies: two of them do not have a Delivery Room.

We obtained a 100% (113/113) response rate to the questionnaire by both physicians and nurses responsible for each Italian NICU.

### Physicians questionnaires

#### Management in the Delivery Room

Sustained Lung Inflation (SLI) was used to support ELGANs in 74.8% of centres, while in 19.1% this strategy was applied upon physician’s preference. In 99 (89.2%) centres, nasal CPAP was used in the DR to assist spontaneously breathing preterm infants with signs of RDS (Table 1).

**Table 1:** Training request, respiratory strategies in the delivery room and NICUs, analgesia from the physicians’ questionnaires

Training Request	Number/Total	%
Need for professional training	102/113	90.2
High-Frequency Oscillatory Ventilation (HFOV)	51/113	45.1
Non Invasive Ventilation (NIV)	63/113	55.7
Conventional Mechanical Ventilation (CMV)	3/113	2.7
Volume-targeted ventilation (VT-CMV)	17/113	15.0
Others	17/113	15.0
Residential Training	102/113	90.2
Management in the Delivery Room		
Sustained Lung Inflation	83/111	74.8
Nasal Continuous Positive Airway Pressure (nCPAP)	99/111	89.2
Ventilatory Support in NICU		
Invasive ventilation		
Conventional Mechanical Ventilation (CMV)	72/113	63.7
High-Frequency Oscillatory Ventilation (HFOV)	24/113	21.2
HFOV and CMV	17/113	15.0
VT-CMV where CMV is used as first intention	31/113	27.4
VT-CMV only during the weaning phase	51/113	45.1
Non invasive ventilation		
nCPAP	113/113	100
Bilevel CPAP	84/113	74.3
High flow nasal cannula (HFNC)	68/113	60.2
Nasal intermittent positive pressure ventilation (NIPPV)	45/113	40.0
Synchronized nasal intermittent positive pressure ventilation (SNIPPV)	10/113	8.8
Nasal HFOV	6/113	5.3
Surfactant Administration in spontaneously breathing infants	Number	%
INSURE approach	103/113	91.1
LISA technique	22/113	19.5
Analgesia and sedation		
Analgesia and sedation during mechanical ventilation	86/113	76.1
Analgesia and sedation during INSURE	61/103	59.2
Analgesia and sedation during LISA	10/22	45.4

#### Invasive Ventilatory support

Conventional Mechanical Ventilation (CMV) was used as first intention for ELGANs in 72 (63.7%) NICUs, while 24 (21.2%) preferred HFOV. Seventeen (15.0%) units used both, indifferently. Among units using preferentially CMV, volume-targeted ventilation (VTV) was commonly chosen during the acute phase of RDS in 31 (27.4%), while in 51 (45.1%) of them this mode was only set during the weaning phase. Analgesia and sedation during mechanical ventilation were used by 76.1% of NICUs, being the fentanyl the most used drug (90%).

#### Surfactant administration for infants in NIV

The INSURE technique in spontaneously breathing ELGANs was currently performed in 91.6% of the centers. Target FiO_2_ to start the procedure was 0.40 in the majority of cases. LISA technique was utilized only in 19.5% of NICUs. Analgesic and sedation procedures for the INSURE approach and LISA technique were reported in 59.2 and 43.5% of centres respectively.

#### Non-invasive ventilation (NIV)

Considering non-invasive techniques, the most popular strategy was nCPAP used by all the Italian NICUs, followed by Bilevel CPAP (74.3%) and High Flow Nasal Cannula (HFNC) (60.2%). Nasal intermittent positive pressure ventilation (NIPPV) was adopted by 40.0% of NICUs, but the synchronized mode (SNIPPV) was used only in 8.8% of them.

### Nurses questionnaires

#### Respiratory settings

Fifty-nine centers (52.2%) used to titrate oxygen therapy within a range of targeted SpO_2_ values; however, these were actually recorded only in 8.0% of nursing charts (Table 2).

**Table 2:** Training requests, respiratory parameters recorded and pain assessment score from the nurses’ questionnaires

	Number/Total	%
Training requests		
Need for professional training	93/113	82.3
High-Frequency Oscillatory Ventilation (HFOV)	32/113	28.3
Non Invasive Ventilation (NIV)	50/113	44.2
Volume-targeted ventilation (VT-CMV)	4/113	3.5
Residential training	104/113	92.0
Target SpO_2_ and respiratory parameters recorded in nursing sheet		
Target SpO_2_ values	9/113	8.0
Respiratory setting	87/113	77.0
CMV respiratory setting	74/85	87.0
CMV measured parameters	60/85	70.5
HFOV respiratory setting	29/37	78.3
HFOV measured parameters recorded	15/37	40.5
NIV respiratory setting recorded	77/113	68.1
NIV spontaneous respiratory rate recorded	54/113	47.8
Silverman score recorded	18/113	15.9
Pain Assessment Score		
Assessment on infants in CMV	35/85	41.2
Assessment on infants in HFOV	32/37	86.5
Assessment on infants in NIV	35/113	30.9

Nurses used to report the respiratory settings on their nursing charts in 77.0% of centers.

Considering CMV, preset respirator settings (Ti, Te, PIP, PEEP, etc.) were reported in 87% of centers, while the measured ones (tidal volume and minute ventilation) in 70.5%.

With regard to HFOV, respiratory set parameters (Hz, MAP, amplitude, etc.) were recorded in 78.3% of the centers, while measured parameters (DCO_2_ and tidal volume) were recorded in 40.5%.

During NIV, respiratory parameters were reported in 68.1% of NICUs, while spontaneous respiratory rate and Silverman score were recorded in 47.8 and 15.9% of the centers, respectively.

#### Pain assessment score

Pain scales were assessed in 41.2% of centers for infants receiving CMV, in 86.5% of centers for infants receiving HFOV and in 30.9% of centers for infants receiving non-invasive respiratory support.

### Need for updating

A relevant percentage of physicians (90.2%) and nurses (82.3%) believed that some aspects of the respiratory support strategies used in their NICU needed to be improved, all of them preferring a residential training on these topics. High-Frequency Oscillatory Ventilation (HFOV) and Non-Invasive Ventilation (NIV) were the most quoted topics that both professionals would like to refine.

## Discussion

This survey provides an insight on ventilatory management of ELGANs in Italy from the delivery room through the NICU. According to the literature’s evidences, routine use of NCPAP in the delivery room is rooted all over Italy with only 10% of centers not observing this approach. Indeed, recent systematic reviews and meta-analysis [8] indicate that avoiding endotracheal intubation at birth in preterm infants reduces the incidence of BPD. Moreover, recent European RDS guidelines recommend stabilizing breathing babies in the delivery room with CPAP of at least 6 cmH_2_O via face mask or nasal prongs [9].

Data on SLI soon after birth for ELGANs in Italy overlap with those reported by Trevisanuto et al. (74.8% vs 76.6%) [10]. Sustained inflation maneuver in DR was widely used in Italy in 2013, before its application was no longer recommended due to lack of evidence of efficacy and safety [11]. In this regard, 2015 resuscitation guidelines suggest to consider SLI only in individual clinical circumstance or research settings until more data on efficacy and safety will be available.

To our knowledge, this is the first Italian survey reporting on ventilatory management of ELGANs in NICUs. Dani et al. [12] described ventilatory management in NICU infants of all gestational ages. Our data show that time-controlled, pressure-limited CMV is still the most used ventilatory strategy for these tiny infants. By contrast, only 27.4% of centers chose the more lung protective VTV during the acute phase of RDS, while 45.1% of them select this mode during the weaning phase, paradoxically when babies are less exposed to lung injury. This occurs despite the wide literature evidence on VTV efficacy in reducing VILI and improving long-term outcomes [13, 14]. We speculate that this can be a consequence of a lack of expertise among physicians and not to the availability of too obsolete machines. As reported by Dargaville and Tingay [4], physicians are not commonly accustomed to consider measured ventilatory parameters in order to tailor the ventilator settings to the specific patient’s needs, which are essential to assure a “protective” invasive ventilation. This underestimation of measured ventilatory parameters is also reflected by the relatively high percentage of NICU nurses (30%) not recording these parameters on their nursing charts. This result highlights the need to raise awareness among both physicians and nurses.

HFOV as first intention is less used than CMV among these particularly delicate preterm infants, but there is a great demand for insights on this topic by both physicians and nurses. The recent introduction in the market of hybrid ventilators able to effectively perform both CMV and HFOV may encourage wider use of HFOV.

Among non-invasive respiratory techniques, NCPAP, Bilevel CPAP and HFNC are the most widely used in our country. Surprisingly, only 40% of centres use NIPPV, despite the emerging evidence that this mode may be superior to NCPAP in preventing extubation failure [15] and treating the acute phase of RDS [16]. Moreover, synchronized NIPPV, that may offer some advantages over NIPPV [17–19], is rarely used probably due to the limited diffusion of ventilators able to deliver it. In general, physicians and nurses of more than 40% of Italian centers claim the need for upgrading their knowledge on non-invasive ventilation.

One hundred and 3 centres (94%) use the INSURE approach for extremely preterm infants suffering from mild to moderate RDS treated with NCPAP as primary mode, indicating that this technique is considered as an useful tool to avoid invasive mechanical ventilation. Less invasive surfactant administration (LISA) technique has been more recently introduced in neonatology and therefore less commonly used around the country.

It is somewhat disappointing the observation that infant’s pain scales are assessed in less than 50% of centers during CMV, even if the percentage increases up to 86.5% during HFOV. Considering that pain management is increasingly recognized as an integral part of effective management of vulnerable babies in the NICU, we believe that analgesic and sedation procedures should be improved in Italian NICUs.

## Conclusions

The results of our study refer to the year 2013 and this certainly represents a limit of our survey, nevertheless they offer the opportunity to focus on the “state of the art” of current practices of neonatal respiratory support for ELGANs in Italian DRs and NICUs. Moreover, they provide information about perceived and not perceived training needs of the medical and nursing staff. Upon these collected data, tailored residential training or courses on ventilatory strategies to be introduced or improved in each unit may represent the preferred way for refining and standardizing ELGANs’ respiratory care in our country. This opportunity may be also relevant in consideration that newer machines and ventilatory techniques are made available by the market and they need an optimal staff training to be properly utilized.

## Abbreviations

BiPAP: Bilevel Positive Airway Pressure
BiPAP: Bi-level positive airway pressure
CMV: Conventional mechanical ventilation
HFNC: High Flow Nasal Cannula
HFNC: High-flow nasal cannula
HFOV: High-frequency oscillatory ventilation
iNO: Inhaled nitric oxide
LISA: Less Invasive Surfactant Administration
MV: Mechanical ventilation
NCPAP: Nasal continuous positive airway pressure
NICU: Neonatal intensive care unit
NIPPV: Nasal intermittent positive pressure ventilation
RDS: Respiratory distress syndrome
SIMV: Synchronized intermittent mandatory ventilation
VTV: Volume Targeted Ventilation
